# Purification of Bone Marrow Clonal Cells from Patients with Myelodysplastic Syndrome via IGF-IR

**DOI:** 10.1371/journal.pone.0140372

**Published:** 2015-10-15

**Authors:** Qi He, Chun-Kang Chang, Feng Xu, Qing-Xia Zhang, Wen-Hui Shi, Xiao Li

**Affiliations:** The Hematology Department of the Sixth Hospital affiliated with Shanghai Jiaotong University, Shanghai, China, 200233; Queen's University Belfast, UNITED KINGDOM

## Abstract

Malignant clonal cells purification can greatly benefit basic and clinical studies in myelodysplastic syndrome (MDS). In this study, we investigated the potential of using type 1 insulin-like growth factor receptor (IGF-IR) as a marker for purification of malignant bone marrow clonal cells from patients with MDS. The average percentage of IGF-IR expression in CD34^+^ bone marrow cells among 15 normal controls was 4.5%, 70% of which also express the erythroid lineage marker CD235a. This indicates that IGF-IR mainly express in erythropoiesis. The expression of IGF-IR in CD34^+^ cells of 55 MDS patients was significantly higher than that of cells from the normal controls (54.0 vs. 4.5%). Based on the pattern of IGF-IR expression in MDS patients and normal controls, sorting of IGF-IR-positive and removal of CD235a-positive erythroid lineage cells with combination of FISH detection were performed on MDS samples with chromosomal abnormalities. The percentage of malignant clonal cells significantly increased after sorting. The enrichment effect was more significant in clonal cells with a previous percentage lower than 50%. This enrichment effect was present in samples from patients with +8, 5q-/-5, 20q-/-20 or 7q-/-7 chromosomal abnormalities. These data suggest that IGF-IR can be used as a marker for MDS bone marrow clonal cells and using flow cytometry for positive IGF-IR sorting may effectively purify MDS clonal cells.

## Introduction

Myelodysplastic syndrome (MDS) is a group of heterogeneous diseases with clonal hematopoietic disorders. One or more lineages of hematopoietic cells could be affected in MDS patients. MDS manifests decreased peripheral blood counts and abnormal morphology. The patients are easily developed to leukemia [1]. Approximately 40–50% patients have abnormal karyotypes [2]. For these patients, fluorescence in situ hybridization (FISH) technology is generally used to identify malignant clonal cells for further study. However, more than half of MDS patients have normal karyotypes (including abnormalities that cannot be discovered using the current karyotype analysis technology). FISH technology cannot be used to distinguish malignant clonal cells from normal cells in these patients. These MDS patients with normal karyotypes still have some abnormalities at the gene level. Identification of malignant clonal cells in these MDS patients with normal karyotypes can greatly benefit basic and clinical studies. Type 1 insulin-like growth factor receptor (IGF-IR) belongs to the tyrosine receptor family. IGF-IR has 70% homology with insulin receptor (IR), which can promote cell proliferation and differentiation. IGF-IR was also found to inhibit cell apoptosis in tumors [3]. High levels of IGF-IR expression are reported in various solid tumors, including breast cancer, colon cancer, prostate cancer, osteosarcoma, and lung cancer [4–8]. IGF-IR is not expressed or has low expression levels in normal bone marrow CD34^+^ cells [9]. IGF-IR mainly has weak functions in the differentiation and maturation of the erythroid lineage, and is not involved in the growth and development of other hematopoietic cells. Our previous study [10] found that bone marrow mononuclear cells (BMNCs) from MDS patients have high levels of IGF-IR expression, and this high expression is more prominent in the high risk group of MDS. In addition, we found that IGF-IR expression have a significant negative correlation with cell apoptosis. Studies on MDS patients with chromosomal abnormalities [11] showed that IGF-IR is primarily expressed on the surface of MDS clonal cells, suggesting that IGF-IR might be a marker for MDS clonal cells. In this study, we tried to investigate if IGF-IR could be a potential tool for the purification of MDS bone marrow clonal cells. After confirming the low expression level of IGF-IR in the early stages of erythroid lineage cells in bone marrow of normal individuals, sorting of IGF-IR-positive MDS bone marrow cells was performed, and the percentage of clonal cells before and after sorting was detected using FISH. Moreover, the IGF-IR specific inhibitor picropodophyllin (PPP) [12] was added in the *in vitro* CD34^+^ cell culture system to observe the changes of clonal cells number at different time points to test if IGF-IR is associated with the growth advantages of clonal cells. Our results showed that IGF-IR may be used as a marker for MDS clonal cells and that sorting for IGF-IR-positive cells may help the partial purification of MDS clonal cells.

## Materials and Methods

### Patients

MDS patients who were diagnosed based on the minimal diagnostic criteria [13] were enrolled in this study. The following classifications were included in this study: the 5q- syndromes, refractory cytopenia with multilineage dysplasia and ring sideroblasts (RCMD-RS), and refractory anemia with excess blasts (RAEB) according to the World Health Organization (WHO) classification [14], the retained chronic myelomonocytic leukemia (CMML) and RAEB in transformation (RAEBt) subtypes as described by the French-American-British classification. Bone marrow specimens of all patients were used for karyotype analyses (G-banding). Chromosomal abnormalities of patients were described according to International System for Human Cytogenetics Nomenclature (ISCN2005) [15]. The detailed information of the patients is shown in Table 1. In addition, 15 healthy volunteers were included as the normal control group.

**Table 1:** The characteristics and IGF-IR purification efficiency in 25 tested patients.

	Sex/age	WHO	Karyotype by G-banding	Clonal cells % in BMNCs before purification(unadjusted value)#	Clonal cells % in BMNCs after CD235a removal	Clonal cells % in BMNCs after purification
**With +8, using the CEP 8 (orange) probe**						
1	M/35	RCMD	47, XY, +8[3]/46,XY[17]	35.5(27.3)		91.3
2	F/48	RCMD	46,XX,+8,der(20;21)(p10;q10)[10]/46,XX[2]	54.3(42.7)		80.0
3	M/17	RCMD	47,XY,+8[25]	83.6(62.7)		96.8
4	F/33	RAEB1	47,XX,+8[18]/46,XX[7]	78.1(59.8)		90.8
5	M/33	RAEB1	46,X,-Y,+der(1)del(1)(p31),+8,-10[10]/46,XY[4]	76.8(60.7)		96.0
6	F/58	RCMD	47,XX,+8[3]/46,XX[22]	25.0(21.6)		90.8
7	F/32	RAEB1	48,XX,+der(1)del(1)(p13),+8[20]	96.1(76.5)		97.8
*8	F/40	RCMD	47,XX,+8[12]/46,XX[13]	65.6(51.0)	74.8	83.7
*9	M/45	RA	46,XY,t(2;2)(p11.2;p25),+8,del(11)(q14q23),der(20;21)(p10;q10)[4]/46,XY[3]	38.5(31.3)	52.0	79.0
*10	M/56	RCMD	46,XY,del(9)(q22q32)[23]/49,XY,+8,+9,+15,t(17;17)(p10;p10)[2]	19.3(16.9)	53.3	86.5
*11	F/28	RCMD	47,XX,del(1)(p22),der(2),del(7)(q31),+8,der(17)[8]/46,XX[10]	85.8(65.5)	80.2	81.1
*12	M/55	RCMD	47,XY,trp(1)(q21q32),+8[25]	83.6(82.5)	88.4	97.6
Mean				**61.9(49.9)**		**89.3**
**With 5q-/-5, using the LSI EGR1 SO/D5S23, D5S721 probe**						
1	F/34	RAEB1	46,XX,del(5)(q13)[25]	85.2(84.9)		98.0
2	M/20	RAEB1-RS	44,XY,-5,der(17),-18,del(20)(q11.1)[17]/46,XY[8]	70.4(63.7)		89.5
3	F/60	RAEB2	46, XX, del(5)(q14q32)[8]	91.1(78.6)		97.5
*4	M/73	RCMD	46,XY,del(20)(q11.2q13.1)[10]/46,XY,del(5)(q15q31)[3]/46,XY[7]	66.8(55.0)	72.0	82.0
Mean				**78.4(70.6)**		**91.8**
**With 20q-/-20, using the LSI D20S108SO probe**						
1	F/60	RT	46,XX,del(20)(q11.2q13.2)[6]/46,XX[19]	80.8(62.6)		94.6
2	M/71	RAEB2	46, XX, del(20)(q12)[10]	96.5(74.5)		98.4
3	M/59	CMML1	45, XY, -7, del(20)(q12)[8]/46, XY, del(20)(q12)[8]/46, XY[5]	66.6(48.6)		67.5
*4	F/62	RCMD	46,XX,der(16)t(1;16)(q21;q12.1),del(20)(q12)[6]	86.6(75.7)	89.0	97.6
*5	M/52	RAEB1	46,XY,del(20)(q11.2q12)[13]/46,XY[12]	79.0(64.1)	79.3	93.5
*6	M/68	RCMD	46,XY,del(20)(q12)[3]	84.8(68.0)	91.1	98.3
*7	M/69	RCMD	46,XY,del(20)(q12q13.2)[15]	90.0(88.1)	86.0	96.6
Mean				**83.5(68.8)**		**92.4**
**With 7q-/-7, using the LSI D7S486SO or CEP7 probe**						
1	F/64	RCMD	45, XX, -7[22] /45, XX[3]	24.7(16.7)		59.8
*2	M/56	RCMD	46,XY,der(7)t(1;7)(q10;q10)[10]/46,XY[4]	85.4(70.5)	89.4	94.1
Total: 25 cases				**70.0(58.0)**		**89.6**

Notes: RCMD, refractory cytopenia with multilineage dysplasia; CMML, chronic myelomonocytic leukemia; RAEB, refractory anemia with excess blasts; RA, refractory anemia; RT, refractory thrombocytopenia

* indicates that the erythroid lineage cells of the patients were first removed before positive IGF-IR sorting

# indicates the initial data which were unadjusted.

### Ethics statement

All subjects provided written informed consent. The written informed consent was obtained from patient himself (if minors/children participants, written informed consent was obtained from their guardians). This study was approved by the Ethics Committee of the Sixth Hospital affiliated with Shanghai Jiaotong University. All patient-relevant research strictly abided by the Declaration of Helsinki.

### Detection of the percentage of IGF-IR-positive cells in all fractions of bone marrow using flow cytometry

Heparinized bone marrow fluids were aliquoted, and flow cytometry antibodies (BD, USA), including CD235a-FITC, CD3-APC, CD19-FITC, CD34-APC, and CD221-PE (IGF-IR), were then added to the fluids. After hemolysis treatment, samples were incubated for 15 min in the dark and subjected to flow cytometry (FACS Calibur) to measure the percentages of IGF-IR-positive cells in the 4 cell fractions. For the detection of each surface marker, at least 1x10^5^ cells were required. All samples were subjected to flow cytometry within 6 h of isolation from the body. The set of tubes was as follows: IgG1a-APC/IgG1a-FITC/IgG1a-PE, CD34-APC/CD221-PE, CD235a-FITC/CD221-PE, CD3-APC/CD221-PE, CD19-FITC/CD221-PE.

### Isolation of BMNCs

Heparinized bone marrow fluid was diluted using Hank's Balanced Salt Solution (HBSS; pH 7.2; without Ca^2+^ and Mg^2+^; Gibco/Life Technologies, Carlsbad, CA, USA) at an equal ratio. After thoroughly mixing, the diluted bone marrow fluid was slowly added into an equal volume of lymphocyte separation fluid (Ficoll; Cedarlane, Burlinton, ON, Canada) for a suspension on the surface of the lymphocyte separation fluid. The sample was centrifuged in a horizontal centrifuge at 1500rpm for 35 min at room temperature. The cloudy BMNC layer on top of the Ficoll layer was collected, washed twice with PBS (pH 7.2; without Ca^2+^ and Mg^2+^; Gibco/Life Technologies), counted, and stored for future usage.

### Sorting of IGF-IR-positive cells in BMNCs using flow cytometry

Isolated mononuclear cells were suspended in 100 μL of PBS at 1×10^7^ cells, and 10 μL of CD221 (IGF-IR) flow cytometry antibody (BD, USA) was added into the cell suspension. The cell suspension was then incubated at room temperature in the dark for 15 min. Samples were washed once with 2 mL of PBS and centrifuged at 1500 rpm for 10 min. The supernatant was discarded, and the cells were resuspended in 500 μL of PBS. Samples were loaded onto a flow cytometer (FACS Aria, BD, USA) for sorting. After sorting, cells were washed once, centrifuged at 1500 rpm for 10 min, resuspended in 1 mL of PBS, and counted. Fetal bovine serum (Sciencell, USA) was added at a ratio of 1:10 (v/v). Cells before and after sorting were smeared onto slides at 1×10^5^ cells/slide. After the slides dried, they were wrapped with aluminum foil and stored at -20°C for subsequent FISH detection.

### Removal of erythroid lineage cells from BMNCs

CD235a-positive BMNCs were sorted using immunomagnetic beads, and CD235a-negative BMNCs were retained. Sorting was performed using a CD235a MicroBead Kit (Miltenyi Biotec, Germany) according to the instruction manual provided by Miltenyi Biotec. Magnetic beads (Multisort CD235a Microbeads) that bind human CD235a were used for sorting, and CD235a-negative cells were obtained using negative selection. CD235a-negative cells were centrifuged and counted. The cell concentration was adjusted using fetal bovine serum at a ratio of 1:10 (v/v), and 1×10^5^ cells were placed onto each slide. After the slides dried at room temperature, they were sealed tightly and stored at -20°C for future FISH detection.

### Separation of CD34^+^ cells using immunomagnetic beads

CD34^+^ cells from 1×10^7^ isolated mononuclear cells were sorted using a CD34^+^ selection kit (EasySep™ Human CD34 Positive Selection Kit, 18056, Miltenyi Biotec, Germany) according to the instruction manual provided by Miltenyi Biotec. The sorting used magnetic beads that bind human CD34 (Multisort CD34 Microbeads) to obtain CD34^+^ cells through positive selection. All manipulations were performed in a sterile workbench with a laminar flow hood.

### Cell culture and PPP drug intervention experiments

Sorted CD34^+^ cells were cultured in 5 mL of stem cell culture medium (StemSpan™ SFEM, 09650, STEMCELL Technologies, Vancouver, BC, Canada), and 10 μL of cytokines (Flt3 ligand, Stem Cell Factor, IL-3 and IL-6)(StemSpan™ CC100) was added for amplification. Cells were cultured for 7–10 days until the cell number reached 5×10^6^ before the intervention experiments were conducted. In preliminary experiments, this concentration of cytokines could make the cell number reach 5×10^6^ after cultured for 10 days, though the cultured cell growth was slower than that in the concentration of cytokines suggested by protocols (S1 Fig).

To observe if IGF-IR is associated with the growth advantages of clonal cells, the IGF-IR specific inhibitor picropodophyllin (PPP) [12] was added in the *in vitro* CD34^+^ cell culture system to observe the changes of clonal cells number at different time points. PPP was purchased from Santa Cruz Biotechnology (USA) and was dissolved in DMSO at a concentration of 0.5mM. The amount of cultured cells was reduced by 30% to 35% at 1μM PPP for 72 hours in preliminary experiment (data not shown), and PPP treatment with 1μM was in favor of our observation on the cultured cells growth for long time. PPP (1 μM) was added to each sample of cultured cells, and an equal volume of DMSO was used as a negative control. At 0, 24, 48, 72, 96, and 168 h (7 d), 1x10^4^ cells were collected for the preparation of smears. Cells were naturally dried at room temperature, sealed, and stored at -20 C for FISH detection. At the same time, some cells were stained by Trypan Blue and the mortality was calculated.

### Detection of abnormal clones using FISH

Fluorescent probes were purchased from Vysis (Abbott Laboratories, Chicago IL, USA), including CEP 8 (orange) probe, LSI EGR1 SO/D5S23,D5S721 probe, LSI D20S108SO probe, LSI D7S486SO and CEP7 probe. For hybridization, the cell smears were removed from storage at -20 C and placed at room temperature for at least 24 h. The smears were then fixed in a 4% formaldehyde solution for 10 min, washed with PBS, digested with pepsin (0.1 mg/mL) at 37°C for 10 min, washed with PBS, and fixed again in a 4% formaldehyde solution for 5 min. Samples were dehydrated in a 70, 85, and 100% ethanol gradient at 2 min per step, and the samples were then dried at 45–50°C, placed in a curing solution at 73 C for 10–20 min, and denatured for 5 min at 75°C in a denature solution. The samples were immediately placed in a 70, 85, and 100% ethanol gradient for dehydration at -20°C, and the samples were then dried. After drying, a DNA probe (10 μL) that had been denatured at 75°C was added immediately in the hybridization area. After the slides were sealed, the hybridization was performed at 37°C in a moisture box for 14–17 h. The slides were washed and dried in the dark. After being dried, the slides were counterstained with 4’,6-diamidino-2-phenylindole (DAPI) containing the anti-fading agent. Based on the chromosomal abnormalities confirmed by G-banding, different targeted probes were selected for different patients. The fluorescence signals were observed with an upright fluorescence microscope (DM5000B; Leica Camera AG, Solms, Germany). Digital signals were captured using a DFC350FX CCD camera and the data were analyzed using Leica CW4000 FISH software.

### Statistical analyses

The distribution of the percentages of abnormal clones in different cell groups was examined using the paired t-test and student’s t-test. SPSS software (version 17.0; Statistical Package of Social Sciences, USA) was used for analysis. P<0.05 indicated statistical significance.

## Results

### Clinical features of patients

In this study, 55 MDS patients (32 males and 23 females) were included, and the median age was 47 (17–80). Twenty-five (14 males and 11 females) of these 55 patients had chromosomal abnormalities, with a median age of 55 (17–73). These 25 samples were all subjected to FISH analysis. Among these patients, 12, 4, 7, and 2 of them showed +8, 5q-/-5, 20q-/-20, and 7q-/-7 abnormalities respectively. The detailed information of these 25 patients, including clinical features, WHO/FAB classification, and karyotypes, is shown in Table 1.

### Expression of IGF-IR in BMNCs in normal controls

**Fig 1:**
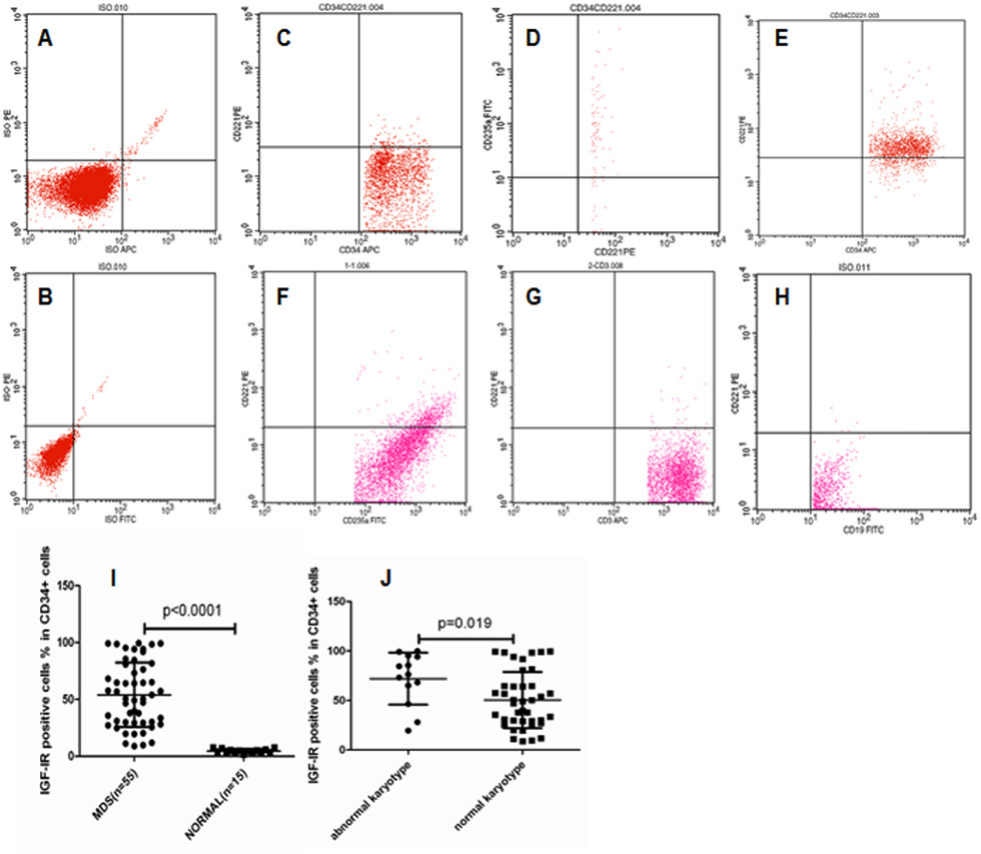
Expression of IGF-IR in CD34+ cells from normal controls and in CD34+ cells, erythroid lineage cells, T lymphocytes, and B lymphocytes from MDS patients. (A) and (B) were isotype controls for the detection. (C) The expression of IGF-IR on CD134+ cells from 1 normal control was 4.3%. (D) Of these CD34+ IGF-IR+ cells, 75.3% expressed the erythroid lineage marker CD235a. (E) The expression of IGF-IR on bone marrow CD34^+^ cells was 82.9% in 1 MDS patient. (F) IGF-IR was expressed on the surface of 15.8% erythroid lineage cells from the same MDS patient. There was not significant expression of IGF-IR on the T lymphocytes (G) or B lymphocytes (H) from the same MDS patient. (I) The expression of IGF-IR of CD34^+^ cells from the 55 MDS patients was significantly higher than that of cells from the normal controls (54.0 vs. 4.5%; *P*<0.0001). (J) The expression of IGF-IR of CD34^+^ cells in the abnormal chromosome group was significantly higher than that in the normal chromosome group (72.0 vs. 50.4%; *P* = 0.019).

IGF-IR expression on CD34^+^ BMNCs was tested in 15 normal controls. The results showed that only 4.5% of CD34^+^ cells from normal controls express IGF-IR. However, more than 70% of these IGF-IR-positive CD34^+^ cells also expressed the erythroid lineage marker CD235a (Fig 1C and 1D). These results suggested that IGF-IR is primarily expressed in some of early hematopoietic cells of the erythroid lineage and is not expressed on the surface of CD34^+^ cells of the non-erythroid lineage in normal bone marrow. In the mid- and late-stage erythroid lineage hematopoietic cells that did not express CD34 (CD34^-^/CD235a^+^), the average percentage of IGF-IR-positive cells was 2.5%. These results were consistent with the previous study [9] showing that IGF-IR is only slightly associated with erythropoiesis.

### Expression of IGF-IR in each fraction of bone marrow nucleated cells in MDS

The detection results of IGF-IR in the 4 fractions of bone marrow nucleated cells, namely CD34^+^ cells, erythroid lineage cells (CD235a-positive), T lymphocytes (CD3-positive), and B lymphocytes (CD19-positive), from 55 MDS patients showed that IGF-IR expression in the CD34^+^ cells from the MDS patients was significantly higher than that in the cells from the normal controls (54.0 vs. 4.5%; *P<*0.0001) (Fig 1E and 1I). The percentage of CD34^+^ cells with IGF-IR expression from the 25 patients with chromosomal abnormalities was significantly higher than that from patients with normal chromosomes (30 cases) (72.0 vs. 50.4%; *P* = 0.019) (Fig 1J). There was no statistical difference in age, gender or WHO/FAB classification from the IGF-IR expression in the CD34^+^ cells of 55 MDS patients. IGF-IR was also expressed on the surface of erythroid lineage cells (CD34-CD235a+) of MDS patients with a percentage of 15.3%, and there was nearly no expression on the surface of T and B lymphocytes (Fig 1F, 1G and 1H).

### Changes in the percentage of clonal cells before and after IGF-IR sorting

IGF-IR was expressed at a certain level on the surface of MDS erythroid lineage cells, and some MDS patients presented significant hyperplasia of the erythroid lineage. Therefore, to exclude the interference of erythroid lineage cells, erythroid lineage cells in some MDS patients with significant erythroid lineage hyperplasia (granulocyte/erythroid ratio less than 1) were removed before sorting experiments. In addition, because IGF-IR was not significantly expressed in the T and B lymphocytes of MDS patients, the statistical data of the percentage of clonal cells before sorting were adjusted to exclude the interference of different lymphocyte ratios in individuals as follows: the adjusted percentage of clonal cells before sorting = unadjusted percentage of clonal cells before sorting / (1—the percentage of lymphocytes). The percentage of clonal cells before sorting mentioned below was the adjusted value.

**Fig 2:**
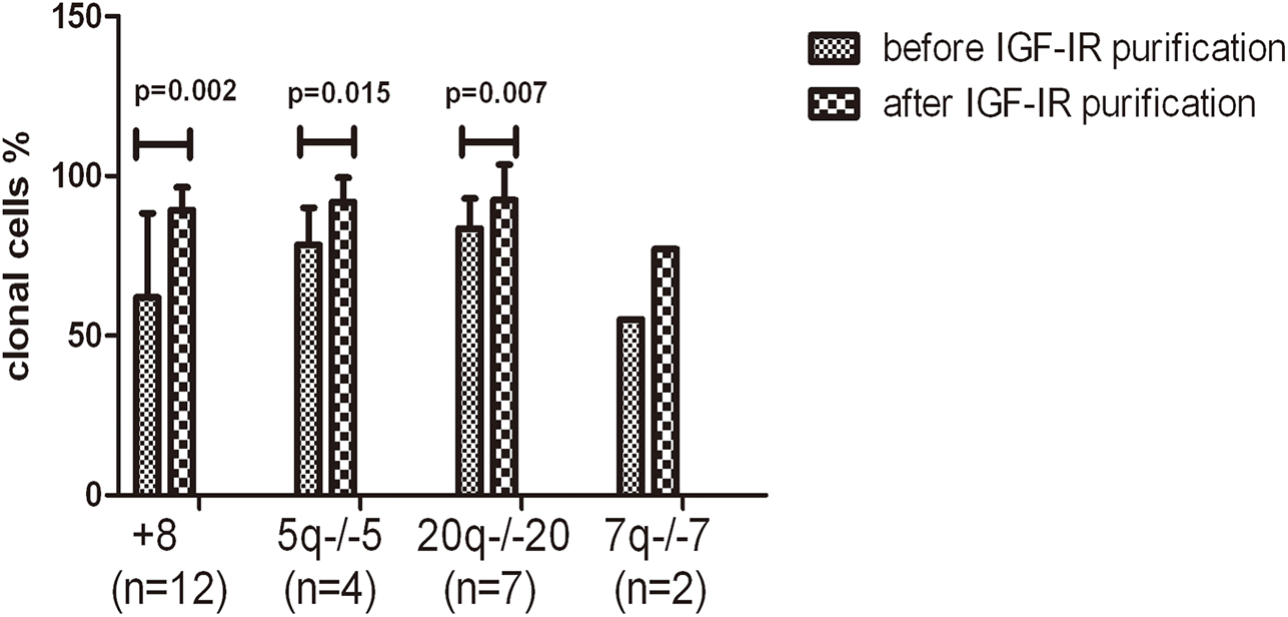
Changes in clonal cells from 25 MDS patients before and after IGF-IR purification, After positive IGF-IR sorting, the percentages of clonal cells in BMNCs from 25 MDS patients significantly increased. In the 4 groups classified based on chromosomal abnormalities (+8, 5q-/-5, 20q-/-20, and 7q-/-7), the percentages of clonal cells after sorting all increased compared to the percentages before sorting. The first 3 groups had statistical significance, and the last group could not be analyzed due to the limited number of cases. The increase in the +8 group was the most significant, and this increase reached 27%.

Cell sorting was conducted on BMNCs from 25 patients with chromosomal abnormalities. Of these cases, 14 cases directly received positive IGF-IR sorting, and the other 11 cases received IGF-IR sorting after the erythroid lineage cells were removed using magnetic beads due to significant erythroid lineage hyperplasia (granulocyte/ erythroid ratio less than 1). The overall levels showed that the percentage of clonal cells in the fractions after IGF-IR sorting was significantly higher than that before sorting (increased from 70.0 to 89.6%; *P*<0.0001). The enrichment of clonal cell that initially had more than 50% clonal cells increased from the average 80.4% to 91.6% (*P*<0.0001). The enrichment effect of the cells that initially had less than 50% clonal cells was more prominent, with the average percentage increasing from 28.6 to 81.5% (*P* = 0.001). Patients were classified into 4 groups (+8, 5q-/-5, 20q-/-20, and 7q-/-7) based on chromosomal abnormalities. All of these 4 groups showed significant increases in the percentage of clonal cells in the fractions after IGF-IR sorting. The first 3 groups all had statistical significant, and the percentage of clonal cells after IGF-IR sorting in the +8 group increased by 27%. Statistical analysis could not be performed on the 7q-/-7 group due to the small case number (Table 1 and Fig 2).

In our previous study [11], fluorescence in situ hybridization (FISH) and immunochemistry (alkaline phosphatase antialkaline phosphatase) were used together to detect the clonal cells and IGF-IR expression in the same MDS patient with known abnormal karyotype. The results from 26 MDS patients showed that IGF-IR expression on clonal cells was markedly elevated compared with that on normal cells in the same MDS patient (*P*<0.0001), and the percentage of clonal cells in IGF-IR positive cells was significantly higher than that in IGF-IR negative cells (85.4 vs. 28.5%; *P*<0.0001) (S2 Fig).

### Changes in the percentage of clonal cells after PPP intervention

**Fig 3:**
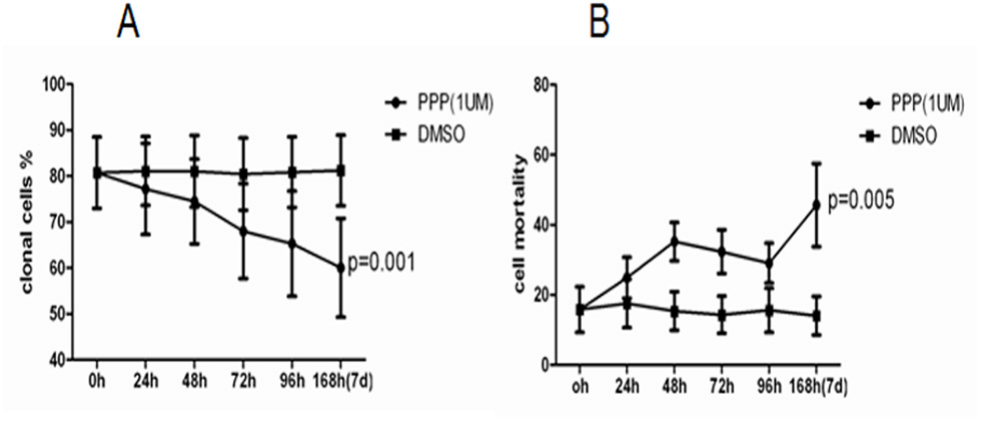
Changes in the cell mortality and the percentages of clonal cells at different time points after PPP intervention. After CD34^+^ cells from 6 MDS patients were amplified *in vitro*, PPP was added at a concentration of 1 μM, and DMSO was used as a control. Cells at 0, 24, 48, 72, 96, and 168 h (7 d) after drug treatment were collected to calculate the mortality by Trypan Blue staining and smeared onto slides to detect the percentage of clonal cells using FISH. Over time, (A) the percentage of clonal cells progressively decreased. The P value resulted from the comparison between before PPP drug intervention (0 h) and 168 h (7 d) after intervention. The average percentage decreased from the initial 80.7 to 60.0%, and the difference was statistically significant. (B) The cell mortality increased over time and the difference from the control was statistically significant.

PPP is a specific inhibitor of IGF-IR and blocks IGF-IR downstream signal transduction [12]. CD34^+^ cells from 6 MDS patients with chromosomal abnormalities were expanded in cell culture. Cells were collected at 0, 24, 48, 72, 96, and 168 h (7 d) after treatment with 1 μM of PPP and the percentage of clonal cells was determined using FISH. Additionally, cell mortality was calculated after trypan blue staining. The results showed that the percentage of clonal cells decreased progressively with the increase of incubation time in PPP containing medium. The average percentage decreased from the initial 80.7% to 60.0% (*P* = 0.001) (Table 2 and Fig 3A). The blank control showed the proportional decrease of both clonal cells and the normal cells in the cultured cells, but did not show a decreasing trend of the percentage of clonal cells. The cell mortality increased over time and the difference from the control was statistically significant (*P* = 0.005) (Fig 3B).

**Table 2:** Changes in the percentages of clonal cells at different time points after PPP intervention *in vitro*.

	Sex/age	WHO	Karyotype by G-banding	Percentage of clonal cells %						
				Before cells culture	After PPP intervention					
					0 h	24 h	48 h	72 h	96 h	7 d
1	M/62	RAEB2	46,XY,del(5)(q13q31)[18]/46,XY[2]	90.5	90.1	91.2	83.3	86.7	83.3	76.4
2	F/55	RA	47,XX,+8[15]	58.6	57.1	41.2	40.0	35.7	33.6	30.2
3	M/73	RAEB	46,XY,del(5)(q15q31),inv(9)(p12q12)[10]	92.7	93.4	93.9	93.3	82.4	80.6	78.5
4	M/17	MDS-U	47,XY,+8[25]	93.8	94.6	92.6	89.9	84.9	85.0	75.6
5	F/34	RAEBt-CMML2	47,XX,+8[12]	92.3	93.6	93.3	88.1	83.1	84.2	77.2
6	F/62	RAEB2	48,X,-X,der(7)t(7;11)(q11q11),+3mar,inc[2]/46,XX[8]	57.1	55.6	50.9	52.1	35.1	25.0	22.2
Mean				80.8	80.7	77.2	74.5	68.0	65.3	60.0

## Discussion

MDS is a clonal disease. Malignant clonal cells, which co-exist in bone marrow with normal hematopoietic cells in MDS patients, need to be selected for further study. Because MDS clonal cells have strong heterogeneity (such as different morphology and different differentiation stages), the effective isolation of malignant clonal cells is difficult. Currently, FISH technology was used as standard method to detect malignant clones in MDS patients. However, this technology has significant limitations. First, this technology can only target patients with confirmed chromosomal abnormalities. Thus, it cannot be effectively applied in more than 50% of MDS patients without chromosomal abnormalities or with failed conventional karyotype analyses. Second, FISH can only target clonal cells for *in situ* detection, and it cannot be used for the purification of clonal cells for functional studies. Finally, the cost of FISH is high and the probe preparation is relatively complex. In addition, it is impossible to prepare probes for all chromosomal fragments.

Using immunohistochemistry and FISH double labeling, our previous studies [10, 11] showed that IGF-IR expression significantly increased in bone marrow nucleated cells in MDS and that the expression was mostly on the surface of clonal cells, thereby suggesting that IGF-IR might be used as a marker for clonal cells in MDS. According to the literature [9, 16, 17], IGF-IR is expressed at a low level in normal hematopoietic cells, and it has a certain function in the proliferation, differentiation, and maturation of erythroid lineage cells. Moreover, IGF-IR is not expressed on the surface of CD34^+^ cells. Our previous studies [10] also found that IGF-IR is expressed on the surface of normal bone marrow cells in MDS. If IGF-IR is highly expressed in normal cells in MDS, it will be difficult to use IGF-IR for the purification of clonal cells. So this study detected IGF-IR expression in bone marrow CD34^+^ cells of normal controls. The results showed that only 4.5% of normal CD34^+^ cells express IGF-IR, which were mostly erythroid lineage precursors of hematopoietic cells. In CD34^-^ CD235a^+^ cells, only 2.5% of these cells had IGF-IR expression. These results suggested that IGF-IR mainly express in erythropoiesis (especially in early erythropoiesis), indicating that IGF-IR is not an indispensable cytokine with multilineage hematopoietic function in hematopoiesis. Therefore, IGF-IR may be a potential tool for clonal purification.

Analysis of IGF-IR expression in MDS bone marrow cells showed that its expression significantly increased on the surface of CD34^+^ cells and that the increase was even more prominent in the abnormal chromosome group, which further confirmed that IGF-IR expression is associated with clonal proliferation. The expression of IGF-IR on the surface of MDS bone marrow erythroid linage cells was significantly higher than that in the normal controls, with 15.3% of the cells expressing IGF-IR. In addition to the function of clonal proliferation of the erythroid lineage, IGF-IR expression might also be affected by the normal residual bone marrow erythropoiesis. Thus, these results suggested that for patients with extremely active erythroid lineage hyperplasia, the method of removal of erythroid lineage before sorting could be used. Studies have shown that [18, 19] IGF-IR is overexpressed in acute and chronic lymphocytic leukemia cell lineages. However, this study showed that IGF-IR is not significantly expressed on the surface of MDS bone marrow T and B lymphocytes. These results further indicated that IGF-IR is only associated with malignant proliferation and that MDS patients do not have malignant proliferation of lymphocytes. In the experiment of the purification of MDS clonal cells using IGF-IR, MDS BMNCs with chromosomal abnormalities were used for positive IGF-IR sorting, and the sorting of some MDS bone marrow cells with significantly active erythroid linage hyperplasia (granulocyte/erythroid ratio less than 1) was performed after the removal of the erythroid lineage. In addition, to exclude the influences of lymphoid lineage cells on the purification experiment, the percentage of clonal cells before sorting was adjusted after the removal of lymphocytes before being compared with the data after sorting. The results showed that whether using direct IGF-IR sorting or sorting after the removal of the erythroid lineage, the percentages of clonal cells after sorting cells from 25 MDS patients with chromosomal abnormalities all significantly increased from 70.0 to 89.6%, which had a significant purification function. The purification effect in patients who had less than 50% clonal cells before sorting was more significant, with the average value increasing from 28.6 to 81.5% (*P* = 0.001). Regardless of the type of chromosomal abnormality or the WHO classification risk (high or low risk), this purification function had statistical significance.

To validate if IGF-IR was associated with malignant clonal proliferation, this study also performed *in vitro* drug intervention experiments. As an isomer of podophyllotoxin, PPP can specifically block the tyrosine phosphorylation function of IGF-IR, thus blocking its downstream signal transduction, without inhibition of other relevant receptors such as insulin receptor (IR)[12, 20]. Bone marrow CD34^+^ cells from MDS were amplified *in vitro* and then co-incubated with PPP. The results showed that the percentage of clonal cells decreased progressively over time in the presence of PPP, suggesting that PPP primarily functions on clonal cells to inhibit the growth of clonal cells. This result indirectly suggested that IGF-IR is mainly expressed on the surface of clonal cells.

Overall, our study showed that IGF-IR is a cytokine that is associated with malignant clonal proliferation in MDS and is not necessary for normal hematopoiesis. Therefore, IGF-IR may be a marker for purification of malignant clonal cells in MDS. Our study also confirmed the purification function of IGF-IR for bone marrow clonal cells in MDS, which suggested that IGF-IR may be used as a marker for clonal cells.

## Supporting Information

S1 Fig Growth of cultured CD34^+^ cells in the different concentration of cytokines in preliminary experiments. In preliminary experiments, the 10ul of cytokines in the cultured cell could make the cell number reach 5×10^6^ after cultured for 10 days, and the 50ul of cytokines made the cell growth reach the equal number after cultured for 7 days. Though the cultured cell growth of 10ul cytokines was slower than that of 50ul cytokines suggested by protocols, the cell number could reach 5×10^6^, the target of cell culture.
(PDF)

S2 Fig Percentage of clonal cells in IGF-IR^+^ cells and IGF-IR^-^ cells from the same MDS patient with known abnormal karyotype. In our previous study, fluorescence in situ hybridization (FISH) and immunochemistry (alkaline phosphatase antialkaline phosphatase) were used together to detect the clonal cells and IGF-IR expression in the same MDS patient with known abnormal karyotype. The results from 26 MDS patients showed that the percentage of clonal cells in IGF-IR positive cells was significantly higher than that in IGF-IR negative cells (85.4 vs. 28.5%; *P*<0.0001)
(PDF)
