# Supplementary material for: Purification of Bone Marrow Clonal Cells from Patients with Myelodysplastic Syndrome via IGF-IR
Source: PLoS One. 2015 Oct 15;10(10):e0140372. doi: 10.1371/journal.pone.0140372 (PMC4607304; doi:10.1371/journal.pone.0140372)
Supplement: S1 Fig — In preliminary experiments, the 10ul of cytokines in the cultured cell could make the cell number reach 5×10^6^ after cultured for 10 days, and the 50ul of cytokines made the cell growth reach the equal number after cultured for 7 days. Though the cultured cell growth of 10ul cytokines was slower than that of 50ul cytokines suggested by protocols, the cell number could reach 5×10^6^, the target of cell culture. (PDF) [file pone.0140372.s001.pdf]

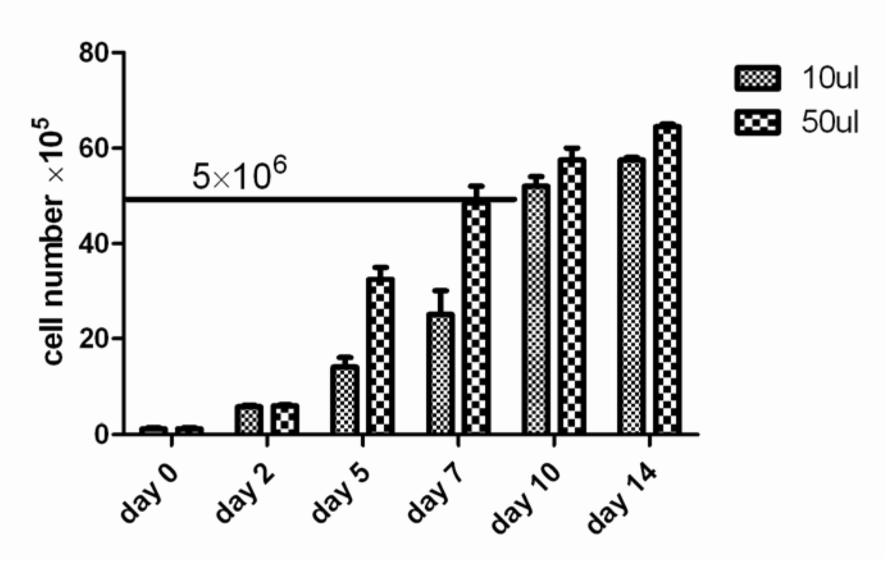

S1 Fig Growth of cultured CD34$^+$ cells in the different concentration of cytokines in preliminary experiments.

In preliminary experiments, the 10ul of cytokines in the cultured cell could make the cell number reach 5×10^6 after cultured for 10 days, and the 50ul of cytokines made the cell growth reach the equal number after cultured for 7 days. Though the cultured cell growth of 10ul cytokines was slower than that of 50ul cytokines suggested by protocols, the cell number could reach 5×10^6, the target of cell culture.
