# Supplementary material for: Purification of Bone Marrow Clonal Cells from Patients with Myelodysplastic Syndrome via IGF-IR
Source: PLoS One. 2015 Oct 15;10(10):e0140372. doi: 10.1371/journal.pone.0140372 (PMC4607304; doi:10.1371/journal.pone.0140372)
Supplement: S2 Fig — In our previous study, fluorescence in situ hybridization (FISH) and immunochemistry (alkaline phosphatase antialkaline phosphatase) were used together to detect the clonal cells and IGF-IR expression in the same MDS patient with known abnormal karyotype. The results from 26 MDS patients showed that the percentage of clonal cells in IGF-IR positive cells was significantly higher than that in IGF-IR negative cells (85.4 vs. 28.5%; P<0.0001) (PDF) [file pone.0140372.s002.pdf]

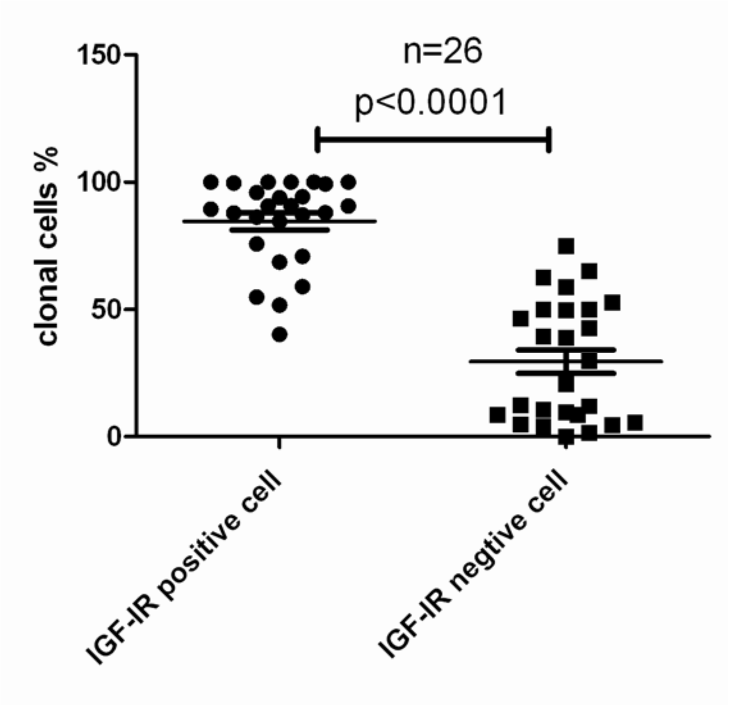

S2 Fig Percentage of clonal cells in IGF-IR^{+} cells and IGF-IR^{-} cells from the same MDS patient with known abnormal karyotype.

In our previous study, fluorescence in situ hybridization (FISH) and immunochemistry (alkaline phosphatase antialkaline phosphatase) were used together to detect the clonal cells and IGF-IR expression in the same MDS patient with known abnormal karyotype. The results from 26 MDS patients showed that the percentage of clonal cells in IGF-IR positive cells was significantly higher than that in IGF-IR negative cells (85.4 vs. 28.5%; $P<0.0001$)
